# Distinct populations of antigen-specific tissue-resident CD8^+^ T cells in human cervix mucosa

**DOI:** 10.1172/jci.insight.149950

**Published:** 2021-08-09

**Authors:** Tao Peng, Khamsone Phasouk, Emily Bossard, Alexis Klock, Lei Jin, Kerry J. Laing, Christine Johnston, Noel A. Williams, Julie L. Czartoski, Dana Varon, Annalyssa N. Long, Jason H. Bielas, Thomas M. Snyder, Harlan Robins, David M. Koelle, M. Juliana McElrath, Anna Wald, Lawrence Corey, Jia Zhu

**Affiliations:** 1 Vaccine and Infectious Disease Division, Fred Hutchinson Cancer Research Center, Seattle, Washington, USA.; 2 Department of Laboratory Medicine and Pathology and; 3 Department of Medicine, University of Washington, Seattle, Washington, USA.; 4 Public Health Sciences Division, Fred Hutchinson Cancer Research Center, Seattle, Washington, USA.; 5 Adaptive Biotechnologies, Seattle, Washington, USA.; 6 Department of Global Health, University of Washington, Seattle, Washington, USA.; 7 Benaroya Research Institute, Seattle, Washington, USA.; 8 Department of Epidemiology, University of Washington, Seattle, Washington, USA.

**Keywords:** Immunology, Virology, Adaptive immunity, T cells

## Abstract

The ectocervix is part of the lower female reproductive tract (FRT), which is susceptible to sexually transmitted infections (STIs). Comprehensive knowledge of the phenotypes and T cell receptor (TCR) repertoire of tissue-resident memory T cells (TRMs) in the human FRT is lacking. We took single-cell RNA-Seq approaches to simultaneously define gene expression and TCR clonotypes of the human ectocervix. There were significantly more CD8^+^ than CD4^+^ T cells. Unsupervised clustering and trajectory analysis identified distinct populations of CD8^+^ T cells with IFNG^hi^GZMB^lo^CD69^hi^CD103^lo^ or IFNG^lo^GZMB^hi^CD69^med^CD103^hi^ phenotypes. Little overlap was seen between their TCR repertoires. Immunofluorescence staining showed that CD103^+^CD8^+^ TRMs were preferentially localized in the epithelium, whereas CD69^+^CD8^+^ TRMs were distributed evenly in the epithelium and stroma. Ex vivo assays indicated that up to 14% of cervical CD8^+^ TRM clonotypes were HSV-2 reactive in HSV-2–seropositive persons, reflecting physiologically relevant localization. Our studies identified subgroups of CD8^+^ TRMs in the human ectocervix that exhibited distinct expression of antiviral defense and tissue residency markers, anatomic locations, and TCR repertoires that target anatomically relevant viral antigens. Optimization of the location, number, and function of FRT TRMs is an important approach for improving host defenses to STIs.

## Graphical abstract

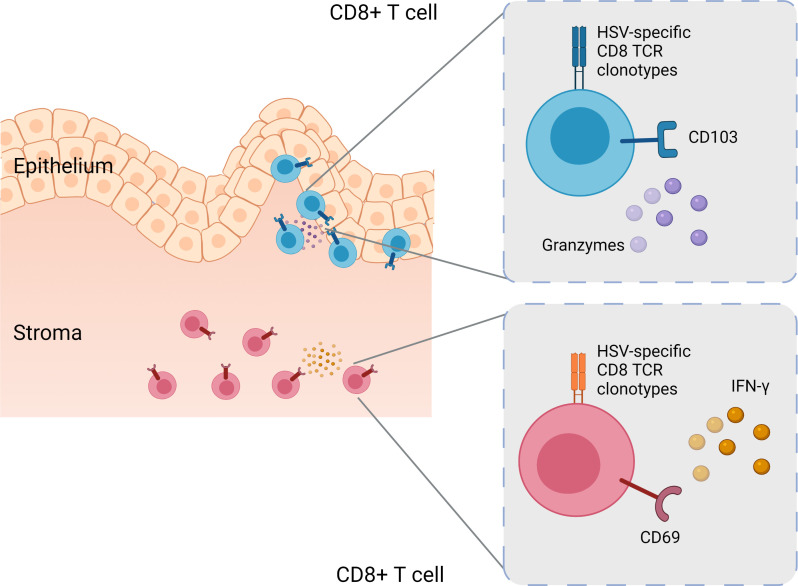

## Introduction

The lower female reproductive tract (FRT) includes the vagina and ectocervix, which are the major entry sites in females for sexually transmitted infections (STIs), including herpes simplex virus type 2 (HSV-2), HIV-1, and certain HPV species. In the case of HSV-2, about 16% of adult women in the United States are HSV-2–seropositive (1, 2), and nearly all have occasional FRT HSV-2 shedding (3, 4). It is generally assumed that the FRT mucosal immune system is critical to protect against STIs. Successful prophylactic HPV vaccines have been developed (5), yet such vaccines for HSV and HIV are currently unavailable. Given that several clinical trials of antibody-based vaccines for HSV-2 have failed to show significant protection (6), it is believed that some combination of CD4^+^ T cells, CD8^+^ T cells, and B cells is necessary to protect against or control HSV-2 infection (7). Our studies using human genital skin biopsies from participants with recurrent HSV-2 infection have shown that CD8^+^ T cells at the dermal-epidermal junction (DEJ CD8) have the hallmarks of tissue-resident memory T cells (TRMs) and that these DEJ CD8 cells likely play critical roles in controlling recurrent HSV-2 infection in the genital skin area (8, 9). Using cervical cytobrush and biopsy specimens, several studies have shown that the human cervix possesses HSV-reactive CD4^+^ and CD8^+^ T cells (10–12). Studies using a mouse vaginal infection model with attenuated HSV-2 mutants have shown that both CD4^+^ and CD8^+^ T cells are necessary to control HSV-2 infection (13, 14). HIV-1–specific cytotoxic CD4^+^ and CD8^+^ T cells localize to the cervix in women infected with HIV (15, 16), and FRT CD4^+^ T cells may mediate HIV infection and transmission (17, 18). It is believed that the FRT CD8 CTL responses offer some protection against HIV-1 infection. However, there is a major knowledge gap in understanding T cell responses at steady-state levels in human cervix mucosa.

The past decade has defined the importance of TRMs as the most abundant memory T cell subset and the first line of defense against pathogens (9, 13, 19–21). TRMs reside in peripheral tissues with limited recirculation, a feature distinguishing them from recirculating effector memory T cells (Tems). T cell subsets are usually distinct and heterogeneous in different peripheral tissue types, such as the small intestine, lung, skin, liver, and FRT (22–24). Transmembrane receptors, including CD69, S1PR1, and CCR7, have been shown to regulate TRM residency (9, 25–27). Integrin molecules, such as CD103 (αEβ7), CD49a (α1β1), and LFA-1 (αLβ2), are known to regulate tissue residency and local immune responses of TRMs. CD103 is enriched in CD8^+^ TRMs in human and mouse skin epidermis (28, 29), CD49a regulates tissue residency of CD8^+^ TRMs in skin and lung tissues (30, 31), and LFA-1 is required for CD8^+^ TRM persistency in liver and lung tissues (32–34). Transcription factors like *TCF7* (*TCF-1*), *KLF2*, *RUNX3*, *ID3*, *ZNF683* (*Hobit*), *PRDM1* (*Blimp1*), *EOMES*, and *TBX21* (*T-bet*) have been shown to regulate tissue residency, memory, and effector functions of TRMs in mouse models (24, 26, 35–38). However, there is a need to discover reliable markers to define T cell subsets in specific human peripheral tissues.

By flow cytometry, CD69^+^ and/or CD103^+^CD4^+^ and CD8^+^ TRMs have been shown to exist in the human cervix and/or other parts of the FRT (11, 39–41). To facilitate vaccine development against pathogens such as HIV and HSV-2, there is a major need to define phenotypes, TCR clonotypes, and anatomic locations of different T cell subsets in human cervix mucosa. We obtained human ectocervical biopsies and sought to define phenotypes and TCR clonotypes of CD8^+^ T cells in the cervix using single-cell immune profiling based on combined gene expression and paired α and β chain TCR sequencing. Our single-cell gene expression analysis revealed 2 distinct populations of CD8^+^ TRMs (cytolytic versus noncytolytic) with differential expression of TRM markers (CD103, CD49a, LFA-1, and CD69). These 2 subsets of CD8^+^ TRMs had distinct anatomic locations and dominant TCR clonotypes in the human cervix. To probe antigenic targets of these CD8^+^ TRM populations among HSV-2–seropositive women, we discovered that up to 14% of the clonotypes from both CD8^+^ TRM subsets in the cervix were shared with HSV-2–reactive CD8^+^ T cell clonotypes in matched blood. These results suggest that different populations of CD8^+^ TRMs are providing distinct defenses against viral infection in the human cervix.

## Results

### Distinct populations of CD8^+^ TRMs with differential expression of tissue residency markers and cytolytic and noncytolytic antiviral genes in human cervix mucosa.

**Figure 1:**
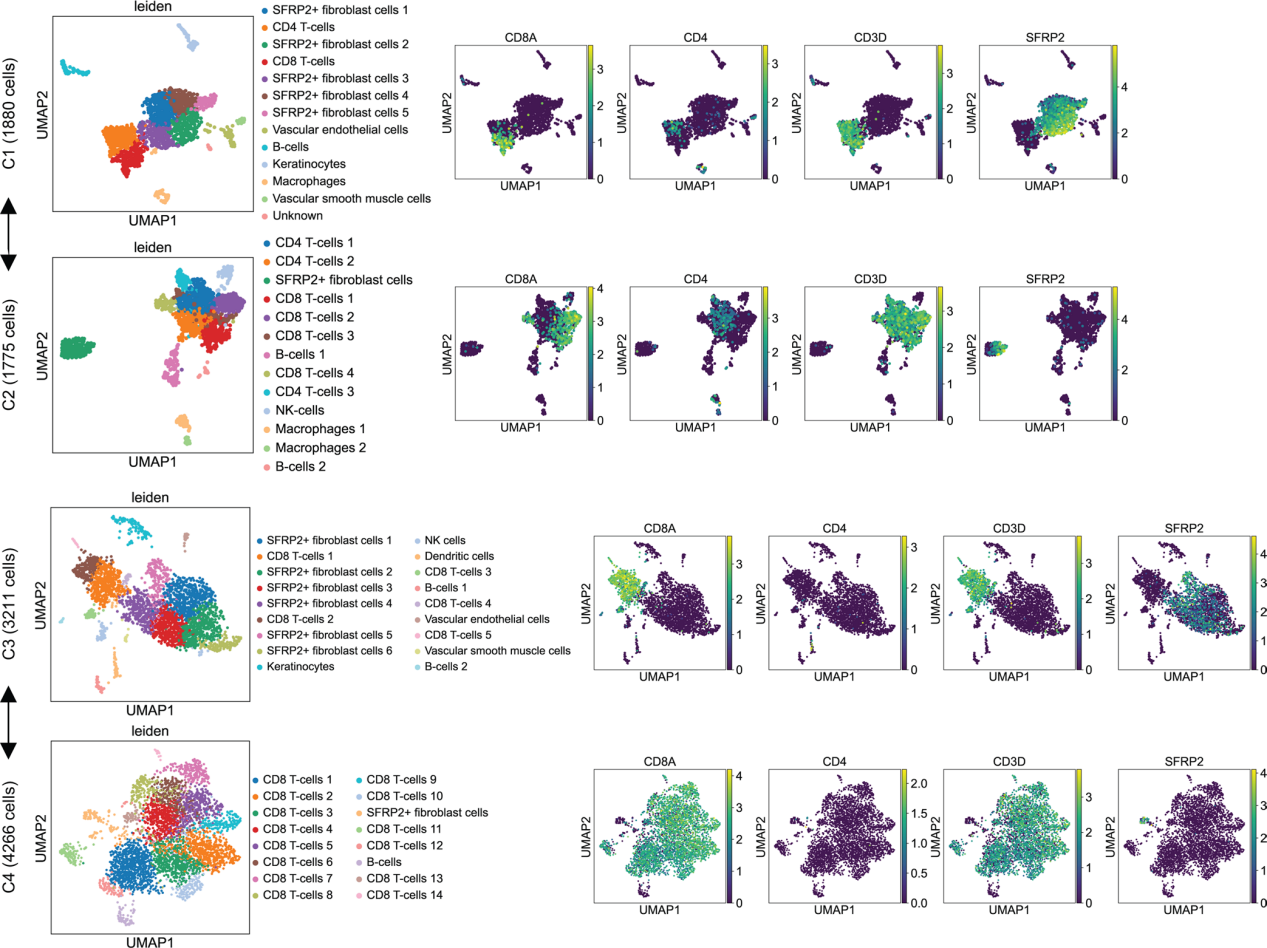
Plating whole single-cell suspension for 1 hour before single-cell RNA-Seq library construction significantly removed fibroblast cells and enriched CD8^+^ and CD4^+^ T cells in the human cervix. CD8A and CD4 are markers for CD8^+^ and CD4^+^ T cells and SFRP2 is a marker for fibroblast cells. CD3D is a marker for both CD8^+^ and CD4^+^ T cells. Comparisons of CD8A, CD4, CD3D, and SFRP2 gene expression in UMAP between C1 (whole suspension) and C2 (whole suspension with 1-hour plating to deplete adherent cells), and between C3 (CD8 negative selection) and C4 (CD8 negative selection with 1-hour plating), are marked by line segments with arrows at both ends.

To define phenotypes and clonotypes of CD8^+^ T cells in the human cervix, we obtained 10 ectocervical biopsies from 8 HSV-2–seropositive individuals, sequenced 23,426 cells for 5′ gene expression, and obtained TCR-vdj data (V, D, and J gene usage, CDR3 sequences, and clonotype frequency [clone count]) from 10,102 cells out of the 23,426 cells (Table 1). We successfully generated single-cell gene expression and TCR-vdj data using 4 methods to process fresh cervical biopsies for sequence library construction: whole suspension, whole suspension with 1-hour plating to remove a significant number of fibroblast cells, CD8 negative selection, and CD8 negative selection with prior 1-hour plating (details in Methods). We used CD8A^+^CD3D^+^, CD4^+^CD3D^+^, and SFRP2^+^ to mark CD8^+^ T cells, CD4^+^ T cells, and fibroblast cells (42), respectively. NK cells were defined as CD8A^lo^CD3D^–^. In both whole suspension and CD8 negative selection, 1-hour plating significantly removed fibroblast cells and enriched CD8^+^ and CD4^+^ T cell populations in the single-cell libraries (Figure 1). Other cell types such as B cells, macrophages, and vascular endothelial cells were found in whole suspension with or without 1-hour plating.

**Figure 2:**
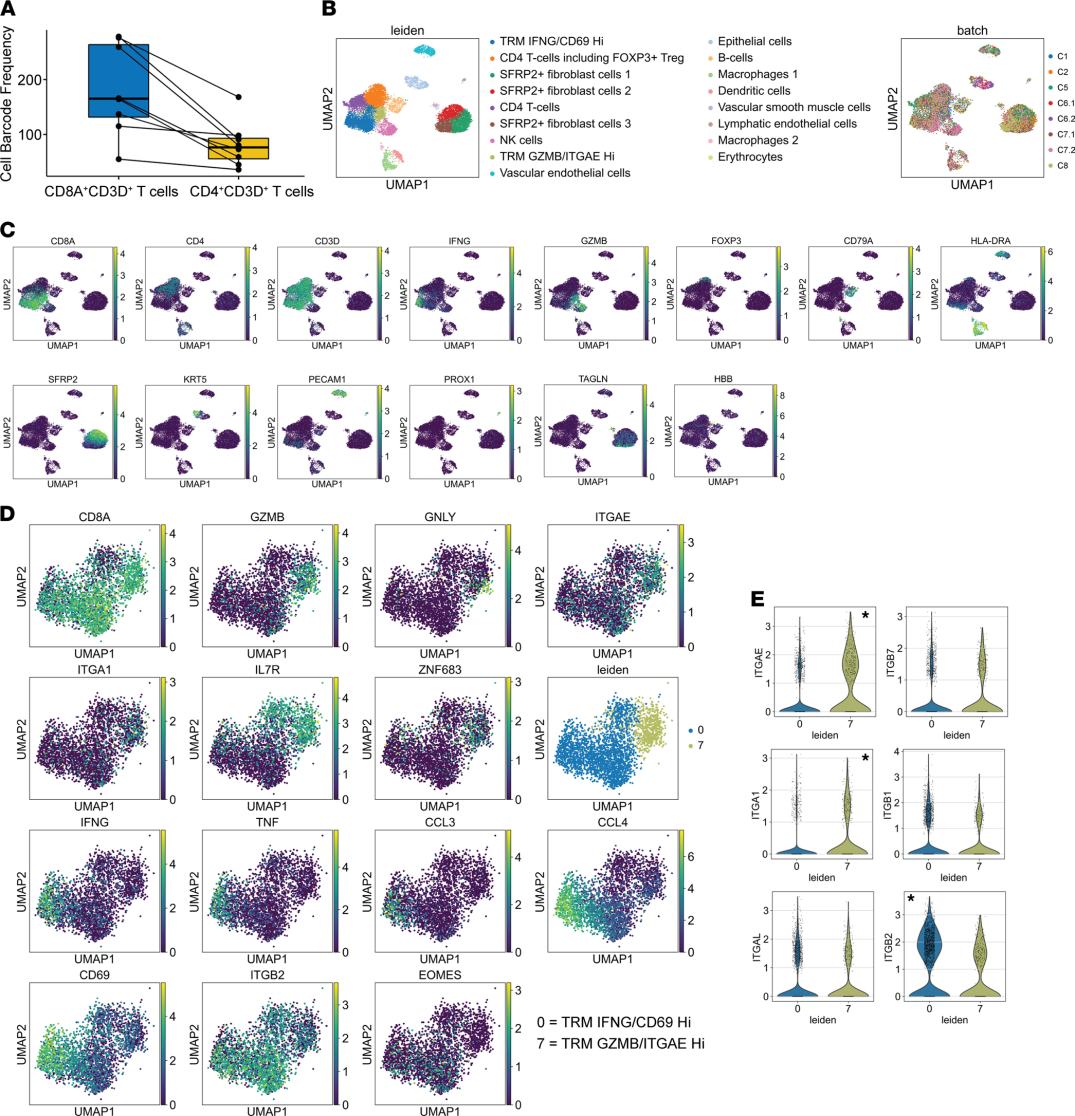
Two subsets of CD8^+^ TRMs with differential expression of tissue residency markers and cytolytic and noncytolytic genes in the human cervix. Eight cervix samples from 6 participants described in Table 1 were used in the analysis. (**A**) More CD8^+^ than CD4^+^ T cells in the human cervix. *P* = 0.003 (paired *t* test, *n* = 8). (**B**) Unsupervised clustering analysis of single-cell gene expression data from the 8 cervical samples identified 17 clusters of cells (left). Contribution of the 8 cervical samples to individual clusters of cells (right). (**C**) UMAP to display gene expression of *CD8A*, *CD4*, *CD3D*, *IFNG*, *GZMB*, *FOXP3*, *CD79A*, *HLA-DRA*, *SFRP2*, *KRT5*, *PECAM1*, *PROX1*, *TAGLN*, and *HBB* in individual clusters of cells. *CD8A*, *CD4*, *CD3D*, *IFNG*, *GZMB* mark T cells and their cytolytic and noncytolytic gene expression; *FOXP3*, *CD79A*, *HLA-DRA*, *SFRP2*, *KRT5*, *PECAM1*, *PROX1*, *TAGLN*, and *HBB* mark Tregs, B cells, macrophages/monocytes/DCs, fibroblast cells, epithelial cells, vascular endothelial cells, lymphatic endothelial cells, vascular smooth muscle cells, and erythrocytes, respectively. (**D**) UMAP to display gene expression of *CD8A*, *GZMB*, *GNLY*, *ITGAE*, *ITGA1*, *IL7R*, *ZNF683*, *IFNG*, *TNF*, *CCL3*, CCL4, *CD69*, *ITGB2*, and *EOMES*. Except *CD8A*, all the other genes were significantly differentially expressed between the 2 clusters of CD8^+^ TRM (*P* < 10^–10^). The IFNG/CD69^hi^ cluster of cells was labeled as TRM IFNG/CD69^hi^ and the GZMB/ITGAE^hi^ cluster of cells was labeled as TRM GZMB/ITGAE^hi^. (**E**) Expression of *ITGAE* and *ITGB7* (CD103), *ITGA1* and *ITGB1* (CD49a), and *ITGAL* and *ITGB2* (LFA-1) in TRM IFNG/CD69^hi^ and TRM GZMB/ITGAE^hi^. *Three genes (*ITGAE*, *ITGA1*, and *ITGB2*) were significantly differentially expressed between the 2 subsets of CD8^+^ TRMs.

Paired *t* tests between the numbers of CD8^+^ T cells (CD8A^+^CD3D^+^) and the numbers of CD4^+^ T cells (CD4^+^CD3D^+^) from the 8 cervical biopsies (C1, C2, C5, C6.1, C6.2, C7.1, C7.2, and C8.1; specimens described in Table 1) suggest that there were significantly more CD8^+^ T cells in the cervix than CD4^+^ T cells (*P* = 0.003) (Figure 2A), which is consistent with previously published enumeration of CD4^+^ and CD8^+^ T cells by flow cytometry in human cervix tissues (43, 44).

Unsupervised clustering analysis of single-cell gene expression data from these 8 cervical samples identified 17 distinct clusters of cells. Analysis of expression of cell type–specific markers across the 17 clusters identified 12 major cell types (CD8^+^ T cells [2 subtypes], CD4^+^ T cells [2 subtypes], NK cells, B cells, macrophages [2 subtypes], DCs, fibroblast cells [3 subtypes], epithelial cells, vascular endothelial cells, lymphatic endothelial cells, vascular smooth muscle cells, and erythrocytes) (Figure 2, B and C). Individual cell types from different cervix samples aligned well within clusters, implying that the difference among the cell types was greater than the biopsy difference (Figure 2B).

Statistical analysis identified significantly expressed genes in individual clusters. CD8^+^ T cells are the major contributors to the expression of IFN-γ, granzyme B (*GZMB*), and perforin (*PRF1*); CD4^+^ T cells dominate the expression of *FOXP3*, a lineage transcription factor for CD4^+^ Tregs, and *CD40LG*, a known molecule that regulates interactions between CD4^+^ T cells and many other cell types, including B cells and DCs (45) (Figure 2C and data not shown). NK cells have low levels of CD8A expression, little CD3D and IFN-γ expression, and high levels of GZMB.

Statistical tests of differentially expressed genes (DEGs) between the 2 clusters of CD8^+^ T cells showed one with significantly higher levels of *GZMB*, *GNLY* (granulysin), *ITGAE* (CD103 α), *ITGA1* (CD49a α), *IL7R* (CD127), and *ZNF683* (Hobit); the other had much higher *IFN-*γ, *TNF* (TNF-α), *CCL3*, *CCL4*, *CD69*, *ITGB2* (LFA-1 β), and *EOMES* (Figure 2D). We labeled the 2 clusters of CD8^+^ T cells as TRM GZMB/ITGAE^hi^ and TRM IFNG/CD69^hi^, respectively. Granzyme B (*GZMB*) and IFN-γ (*IFNG*) encode important cytolytic and noncytolytic T cell effector molecules, respectively. *ITGAE*, *ITGA1*, and *ITGB2* encode the α subunits of CD103 and CD49a and the β subunit of LFA-1, respectively. These 3 integrin molecules are known to play roles in the establishment of T cell tissue residency in peripheral tissues. *ZNF683* is a transcription factor known to regulate tissue residency of T cells, *IL7R* and *EOMES* play critical roles in generation of memory T cells, and *GNLY* encodes a cytotoxic molecule (46). Further analysis of expression of both α and β subunits of CD103, CD49a, and LFA-1 in TRM IFNG/CD69^hi^ and TRM GZMB/ITGAE^hi^ indicated that CD103 αE (*ITGAE*) and CD49a α1 (*ITGA1*) but not LFA-1 αL (*ITGAL*) were significantly higher in TRM GZMB/ITGAE^hi^, while LFA-1 β2 (*ITGB2*) but not CD103 β7 (*ITGB7*) or CD49a β1 (*ITGB1*) were significantly higher in TRM IFNG/CD69^hi^ (Figure 2E). In summary, the data suggest that there are 2 distinct populations of CD8^+^ TRMs in the human cervix with differential expression of tissue residency markers and cytolytic and noncytolytic antiviral genes.

**Figure 3:**
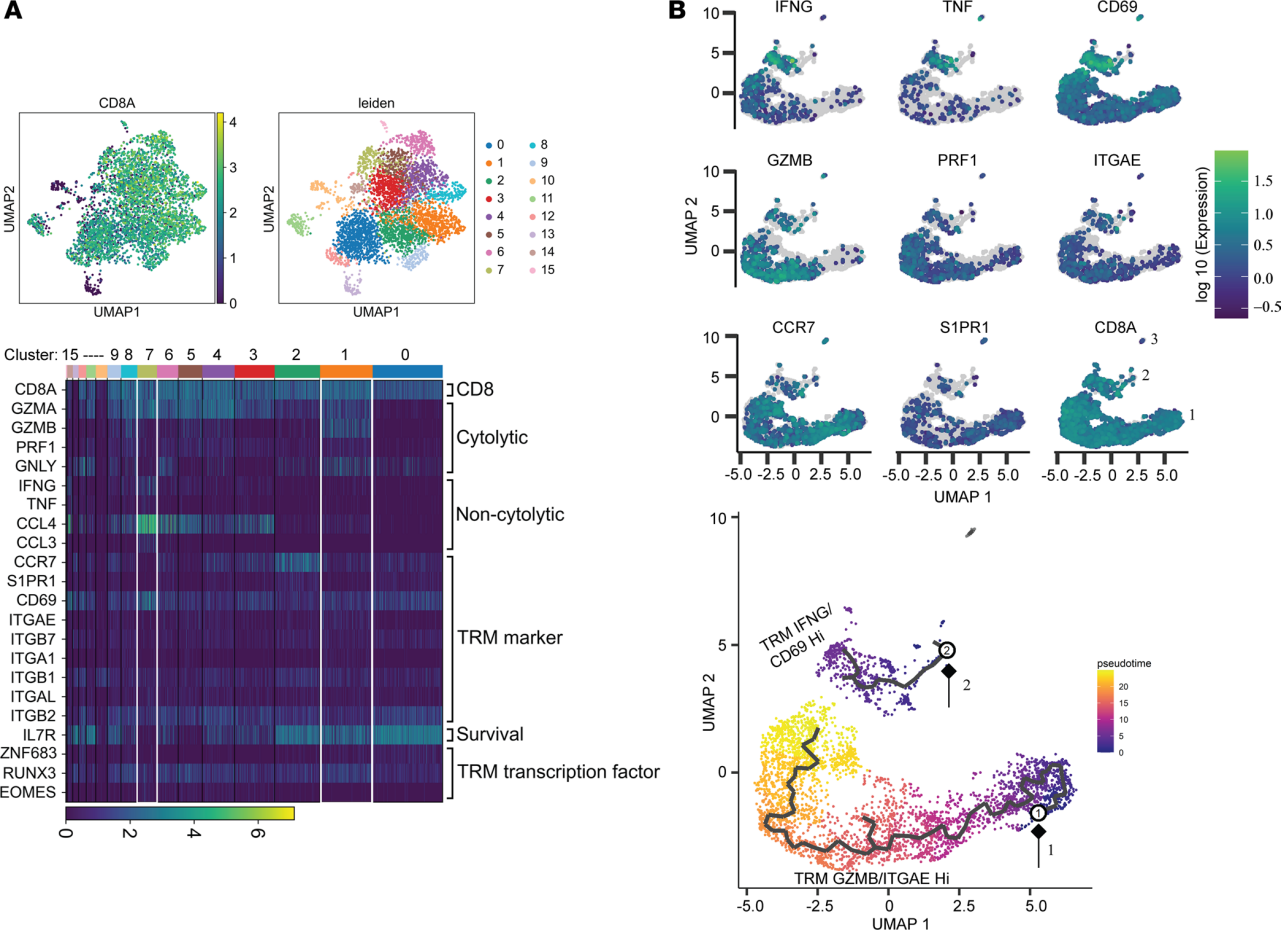
Trajectory analysis of purified CD8^+^ T cells in the human cervix. (**A**) Clustering analysis of single cells. Top panel: expression of *CD8A* in different clusters; clusters 1 and 7 are marked and labeled as TRM GZMB/ITGAE^hi^ and TRM IFNG/CD69^hi^, respectively. Bottom panel: heatmap to show gene expression of functional categories (CD8, cytolytic, noncytolytic, TRM marker, survival, and TRM transcription factor) in different clusters. (**B**) Trajectory analysis of single cells. Top panel: gene expression of individual cells in partition. Genes for *IFNG*, *TNF*, *CD69*, *GZMB*, *PRF1*, *ITAGE*, *CCR7*, *S1PR1*, and *CD8A* are shown. Bottom panel: trajectory analysis in pseudo time. Two trajectory curves were generated with 2 root nodes marked in partitions 1 and 2. The results shown in **A** and **B** were generated from CD8^+^ T cells, which were negatively selected from the cervical sample C4.

To further define potentially different populations of CD8^+^ T cells in the cervix, we purified CD8^+^ T cells using CD8-negative immunomagnetic selection. We generated single-cell gene expression data for 4266 cells in which 3491 cells had detectable CD8A expression in 1 cervix sample (C4). Unsupervised clustering analysis identified 16 clusters of cells, suggesting that CD8^+^ T cells in this cervical specimen had different subtypes (Figure 3A). To define the subtypes of CD8^+^ T cells in the cervix, we chose well-characterized genes in categories of cytolytic (*GZMA*, *GZMB*, *PRF1*, and *GNLY*), noncytolytic (*IFNG*, *TNF*, *CCL4*, and *CCL3* ), tissue residency markers (*CCR7*, *S1PR1*, *CD69*, *ITGAE*, *ITGB7*, *ITGA1*, *ITGB1*, *ITGAL*, and *ITGB2*), T cell survival (*IL7R*), and transcription factors known to regulate T cell tissue residency (*ZNF683*, *RUNX3*, and *EOMES*). *CD69* is an activation marker for T cells and a TRM marker in peripheral tissues, and all the CD8^+^ T cells had moderate to high levels of *CD69* expression; cells in cluster 7 had the highest *CD69* expression (Figure 3A). Cells in cluster 7 also had the highest IFN-γ expression; high levels of *GZMA* yet low levels of *GZMB* and *GNLY*; and low levels of *CCR7* and *S1PR1*, 2 receptors that are known to regulate T cell exit from tissue (26, 47, 48). They also had high levels of *ITGAL* and *ITGB2* (α and β subunits of LFA-1) yet low levels of *ITGAE*, *ITGA1*, and *IL7R*. The group of CD8^+^ T cells in cluster 7 likely represents a type of TRM for immunosurveillance. We labeled them as IFN-γ^hi^ and CD69^hi^ TRM (TRM IFNG/CD69^hi^ as described in Figure 2D). In contrast, cells in cluster 1 had the highest *GZMB*, *ITGAE*, and *IL7R* yet low levels of *IFN-*γ expression; they also expressed moderate levels of *CCR7* and *S1PR1*. We labeled them as GZMB^hi^ and ITGAE^hi^ TRM (TRM GZMB/ITGAE^hi^ as described in Figure 2D). Consistent with the results described in Figure 2D, *ZNF683* (*Hobit*) and *RUNX3* were higher in TRM GZMB/ITGAE^hi^ and EOMES was higher in TRM IFNG/CD69^hi^. In contrast, CD8^+^ T cells in cluster 0 had little expression of cytolytic granzymes (*GZMA*, *GZMB*, and *PRF1*) or *IFN-*γ, yet they had high levels of expression of *CCR7*, *S1PR1*, and *IL7R* but little expression of *ITGAE*, *ITGA1*, or *ITGAL* (Figure 3A). CD8^+^ T cells in cluster 0 appeared to be a type of cells with the potential to exit the cervix. Cells in the other clusters had mixed gene expression patterns of these groups of genes, suggesting that CD8^+^ T cells in the human cervical tissues are heterogeneous. To define the relationship of cells in different clusters, we performed trajectory analysis of the entire group of cells. Clustering analysis partitioned cells into 3 groups, and expression patterns of individual genes (*IFNG*, *TNF*, *CD69*, *GZMB*, *PRF1*, *ITAGE*, *CCR7*, and *S1PR1*) in partition showed that CD8^+^ TRMs with high levels of *IFNG*, *TNF*, and *CD69* were grouped in the left end of partition 2; CD8^+^ TRMs with high levels of *GZMB*, *PRF1*, and *ITGAE* were grouped in the middle section of partition 1; and those with high levels of *CCR7* and *S1PR1* in the right end of partition 1 (Figure 3B). By setting root nodes at the right ends of partitions 1 and 2, 2 trajectory curves placed cells in partitions 1 and 2 in pseudo time, a measure of how CD8^+^ T cells in the human cervix relate to one another biologically (Figure 3B). Detailed trajectory analysis of purified CD8^+^ T cells from the cervix indicated that TRM IFNG/CD69^hi^ and TRM GZMB/ITAGE^hi^ are distinct populations of TRMs in the human ectocervix.

### Distinct anatomic location of different populations of CD8^+^ TRMs in human cervix mucosa.

**Figure 4:**
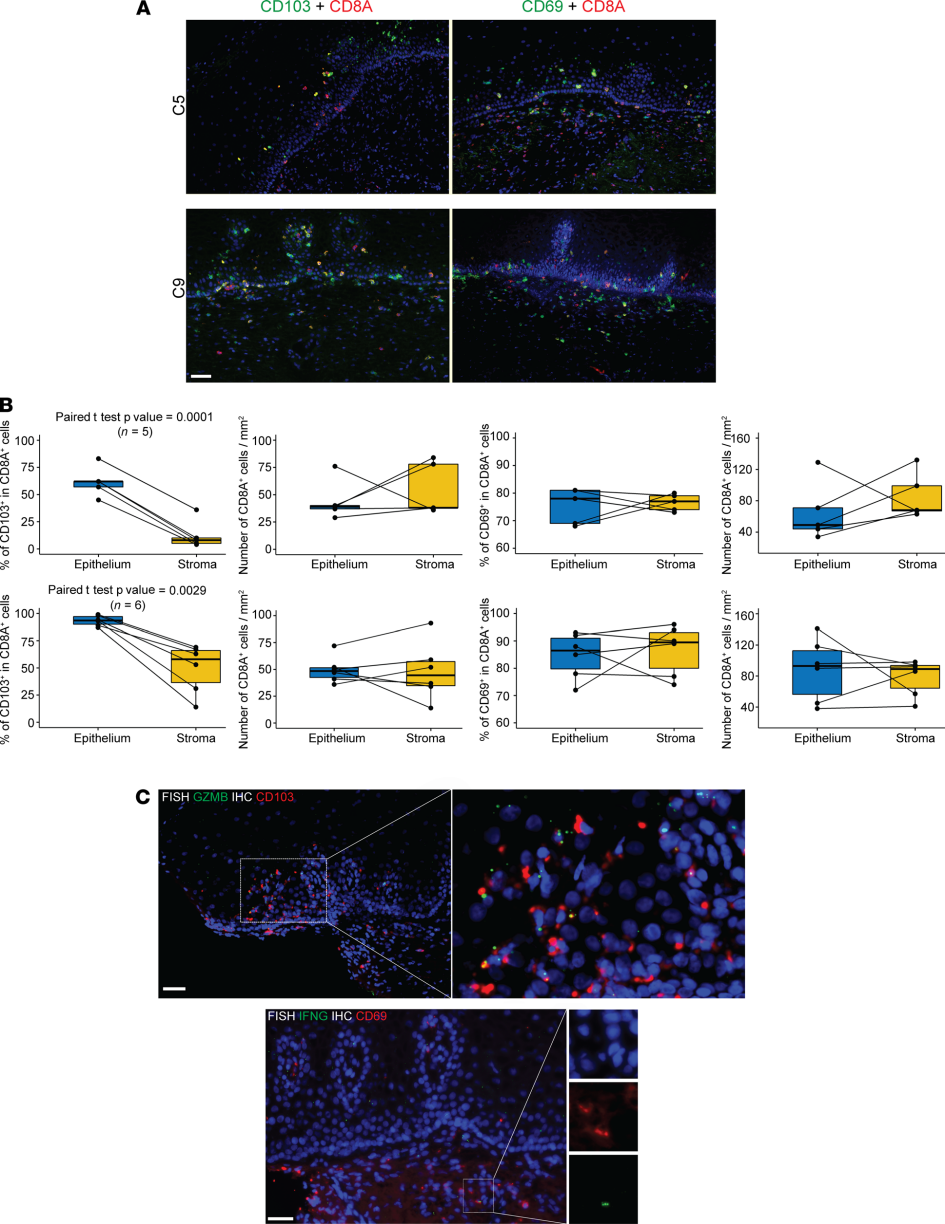
Distinct anatomic location of CD103^+^GZMB^+^CD8A^+^ and CD69^+^IFNG^+^CD8A^+^ T cells in the human cervix. (**A**) Double immunofluorescence staining of cervix biopsies (C5 and C9) with CD8A and CD103 antibodies or CD8A and CD69 antibodies. (**B**) Quantitation of CD103^+^CD8A^+^ and CD69^+^CD8A^+^ cells and density of CD8A^+^ cells in epithelium and stroma from individual cervix biopsies (top row, *n* = 5, C1, C5, C7.2, C8.1, and C8.2 from HSV-2–seropositive participants; bottom row: *n* = 6, C9 to C15 from HSV-2–seronegative participants). (**C**) CD103^+^GZMB^+^ cells in epithelium and CD69^+^IFNG^+^ cells in stroma of cervix biopsies (*n* = 3). Top panels: RNA FISH for GZMB and immunofluorescence staining for CD103; bottom panels: RNA FISH for IFNG and immunofluorescence staining for CD69. Scale bar: 50 μm.

To verify in vivo distribution of 2 CD8^+^ TRM subsets in the human cervix described in Figures 2 and 3 and to confirm that these cell populations were also found in HSV-2–seronegative women, we obtained a separate set of 8 ectocervix biopsies from 7 participants plus saved portions of 4 cervix biopsies, which were used for single-cell immune profiling (Table 2). Five cervix biopsies (C1, C5, C7.2, C8.1, and C8.2) were from HSV-2–seropositive individuals, and 7 biopsies (C9 to C15) were from HSV-2–seronegative individuals. We performed double immunofluorescence staining using CD8A and CD103 antibodies or CD8A and CD69 antibodies to analyze anatomic locations of CD8A^+^CD103^+^ T cells or CD8A^+^CD69^+^ T cells in these cervix samples. Most CD8A^+^CD103^+^ T cells were located in the epithelium, whereas CD8A^+^CD69^+^ T cells were distributed evenly from the epithelium to underlying stroma area (Figure 4A and Supplemental Figures 1 and 2; supplemental material available online with this article; https://doi.org/10.1172/jci.insight.149950DS1). We did the double staining with 11 of the 12 cervix samples and counted the number of CD8A^+^, CD8A^+^CD103^+^, and CD8A^+^CD69^+^ T cells in the epithelium and stroma. On average for the 5 HSV-2–seropositive samples, 62% and 12% of CD8A^+^ T cells were CD103^+^ in the epithelium and underlying stroma, respectively, and this difference was statistically significant (*P* = 0.0001, paired *t* test, *n* = 5) (Figure 4B, top row). For the 6 HSV-2–seronegative samples, the difference was also significant (93% versus 49%, *P* = 0.0029, paired *t* test, *n* = 6) (Figure 4B, bottom row). In contrast, similar percentages of CD8A^+^ T cells were CD69^+^ in the epithelium and stroma (75% versus 77% for HSV-2–seropositive and 85% versus 87% for HSV-2–seronegative). Densities of CD8A^+^ T cells were similar in both areas (Figure 4B). The combination of RNA FISH for GZMB or IFNG and IHC for CD103 or CD69 demonstrated GZMB^+^CD103^+^ cells in the epithelium and IFNG^+^CD69^+^ cells in the underlying stroma area of the cervix biopsy tissues (*n* = 3) (Figure 4C). The distinct anatomic locations of CD8A^+^CD103^+^GZMB^+^and CD8A^+^CD69^+^IFNG^+^ T cells in the human cervix suggest that the 2 CD8^+^ TRM subsets (IFN-γ^hi^CD69^hi^CD103^lo^ versus GZMB^hi^CD103^hi^CD69^med^) revealed by the single-cell gene expression analysis may have different anatomic locations in the human cervix.

### The 2 CD8^+^ TRM subsets have distinct expanded clonotypes in human cervix mucosa.

**Figure 5:**
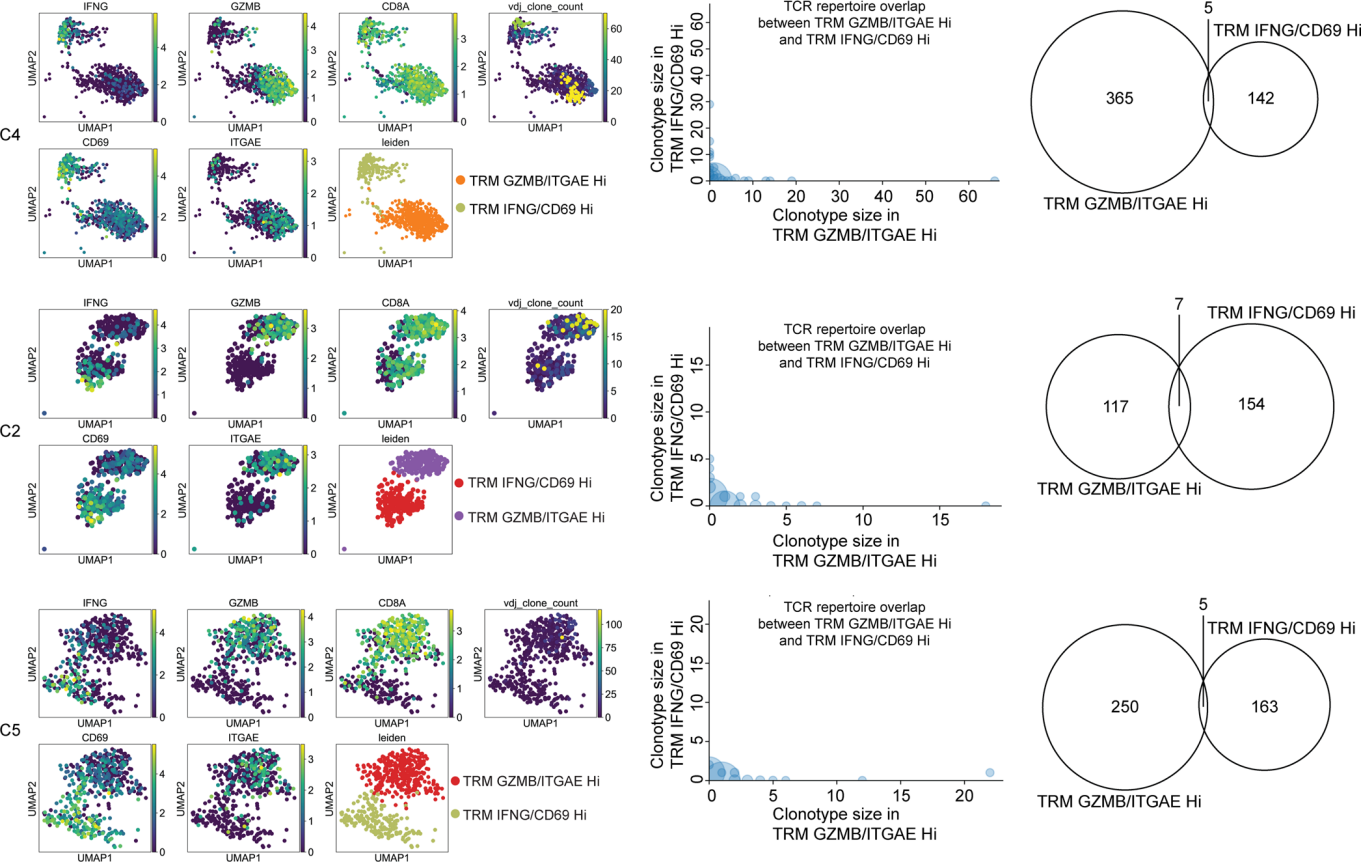
Single-cell TCR clonotype analysis of the human cervix. Top, middle, and bottom rows are from C4, C2, and C5 cervical samples, respectively. Left: display of vdj clone count and expression of 5 genes (*CD8A*, *IFNG*, *CD69*, *GZMB*, and *ITGAE*) in the 2 clusters of cells representing TRM IFNG/CD69^hi^ and TRM GZMB/ITGAE^hi^ in UMAP. VDJ clone count refers to the number of cells that share the same TCR clonotype. Middle: graphs to show TCR clonotype size (number of cells sharing the same clonotype) and TCR clonotype overlaps between TRM GZMB/ITGAE^hi^ and TRM IFNG/CD69^hi^. Individual circles represent individual clonotypes that are shared between the 2 subsets of CD8^+^ TRMs; Half circles represent individual clonotypes that are unique to TRM GZMB/ITGAE^hi^ (*x* axis) or TRM IFNG/CD69^hi^ (*y* axis). Right: Venn diagrams show clonotype overlaps between TRM GZMB/ITGAE^hi^ and TRM IFNG/CD69^hi^. The 2 numbers in the middle of circles in the Venn diagrams refer to numbers of unique clonotypes associated with TRM GZMB/ITGAE^hi^ and TRM IFNG/CD69^hi^. The numbers in the middle of the Venn diagrams refer to numbers of shared TCR clonotypes between TRM GZMB/ITGAE^hi^ and TRM IFNG/CD69^hi^.

We then analyzed the distribution of TCR clonotypes in the 2 clusters of CD8^+^ TRMs (IFNG/CD69^hi^ and TRM GZMB/ITAGE^hi^) from 3 cervical samples (C4, C2, and C5). Both types of TRMs were oligoclonal; however, the TRM GZMB/ITAGE^hi^ subset had higher levels of clonal expansion (Figure 5). For C4, 56 clonotypes out of the 365 unique clonotypes from TRM GZMB/ITAGE^hi^ were detected in more than one cell (expanded), while 19 of the 142 clonotypes in the TRM IFNG/CD69^hi^ subset were expanded. There was no overlap between these expanded clonotypes (Figure 5). Only 5 clonotypes were shared between TRM GZMB/ITAGE^hi^ and TRM IFNG/CD69^hi^ (Figure 5). For C2, 26 clonotypes out of the 117 unique clonotypes from TRM GZMB/ITAGE^hi^ and 17 clonotypes out of the 154 unique clonotypes from TRM IFNG/CD69^hi^ were expanded, and these 2 groups of expanded clonotypes did not have any overlap either (Figure 5). Only 7 singletons from the 154 unique clonotypes from TRM IFNG/CD69^hi^ were shared with the 117 unique clonotypes from TRM GZMB/ITAGE^hi^ (Figure 5). For C5, 4 out of the 32 expanded clonotypes plus 1 singleton from TRM GZMB/ITAGE^hi^ were shared with 5 singletons in TRM IFNG/CD69^hi^ (Figure 5, bottom row). The distinct expanded clonotypes of TRM IFNG/CD69^hi^ and TRM GZMB/ITAGE^hi^ in the 3 cervix samples suggest that the 2 subsets of TRMs described in Figures 2 and 3 had different roles in host defense and separate prior histories responsible for their placement.

### Expanded clonotypes are largely from CD8^+^ T cells, and clonally expanded T cells exhibit high levels of gene expression for granzymes and chemokines in human cervix mucosa.

**Figure 6:**
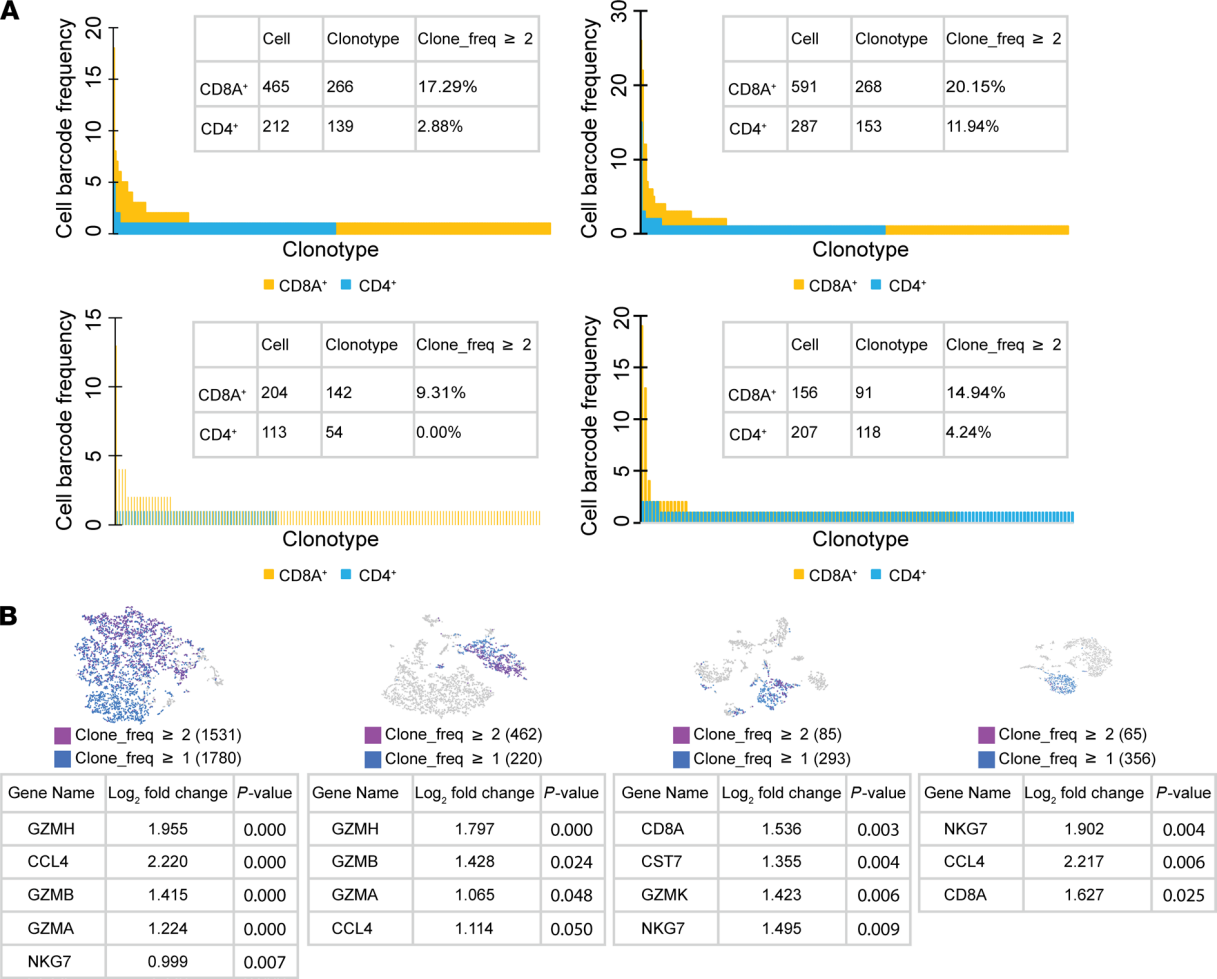
The expanded clonotypes in the human cervix were weighted toward CD8^+^ T cells. (**A**) Frequency of individual clonotypes in CD8A^+^ and CD4^+^ T cells from the 4 cervix samples (top row: C2 and C5; bottom row: C1 and C6.1). *X* axis: individual clonotypes. *Y* axis: cell barcode frequency for individual clonotypes. The tables in individual graphs show numbers of cells that were CD8A^+^ or CD4^+^, numbers of unique TCR clonotypes, and percentages of clonotypes with a frequency of at least 2. (**B**) Differential gene expression between T cells with clonotype frequency of at least 2 and those with singletons in the 4 cervical samples (C4, C3, C6.1, and C1). Top graphs: distribution of clonotypes with frequency of at least 2 and those with singletons in tSNE plots. Bottom tables: lists of genes differentially expressed between T cells with clonotype frequency of at least 2 and those with singletons (*P* ≤ 0.05).

We selected CD8A^+^ and CD4^+^ cells and identified their clonotypes in the 4 cervical samples (C2, C5, C1, and C6.1). In all except C6.1, CD8^+^ T cells had more diverse TCR repertoires than did CD4^+^ T cells. In all 4 cervical samples, CD8^+^ T cells had higher percentages of expanded clonotypes (clonotype frequency ≥ 2) (Figure 6A). The results suggest that expanded clonotypes of T cells in the human cervix were mostly from CD8^+^ T cells.

Since CD8^+^ T cells in the cervix appeared to be the major contributors to cytolytic and noncytolytic gene expression and expanded TCR clonotypes in the human cervix, we evaluated gene expression differences between cells with expanded clonotypes (clonotype frequency ≥ 2) and those with singletons (clonotype frequency = 1). In specimen C4, we obtained gene expression data from more than 4000 cells and TCR-vdj data from more than 3000 cells. Comparison of gene expression between 1531 cells with clonotype frequency of 2 or more and 1780 cells with singletons showed that *GZMH*, *GZMB*, *GZMA*, *NKG7* (a gene encoding a granule protein), and *CCL4* were significantly higher in cells with expanded clonotypes. Comparison of gene expression between 462 cells with expanded clonotypes and 220 cells with singletons from C3 showed that the same set of genes except NKG7 (*GZMH*, *GZMB*, *GZMA*, and *CCL4*) were significantly higher in cells with expanded clonotypes (Figure 6B). We found similar results in 2 additional cervix samples (Figure 6B). These results suggest that clonally expanded T cells possessed higher levels of gene expression for cytotoxicity and chemokines than did TCR singleton T cells.

### Both CD8^+^ TRM subsets in the human cervix target HSV-2 antigens.

The cervix is a site of HSV-2 infection and chronic shedding. Previous work has recovered HSV-2–specific T cells from this site (10, 11). To determine whether the T cell clonotypes associated with 2 distinct populations of CD8^+^ TRM (TRM GZMB/ITAGE^hi^ and TRM IFNG/CD69^hi^ as described in Figures 2–5) in the human cervix were reactive with a local infectious pathogen, we identified HSV-2–reactive TCR β (TCRB) sequences in the blood and sought TCR overlap with cervical T cells. HSV-2–reactive CD8^+^ T cells were purified from blood using expression of CD137 as an activation-induced marker after cross-presentation of HSV-2 by autologous monocyte-derived DCs (49). Genomic DNA prepared from sorted CD137^hi^ CD8^+^ T cells was used for bulk TCRB CDR3 sequencing in a quantitative platform in which productive TCRB CDR3 reads correspond to individual cells (50). We compared such blood TCRB CDR3 sequences from 3 HSV-2–seropositive participants (Pt4, Pt5, and Pt7) with the clonotypes associated with TRM GZMB/ITAGE^hi^ and TRM IFNG/CD69^hi^ in matching cervical specimens (C4, C5, and C7.1). A matched TCRB clonotype is defined as matched TCRB CDR3 amino acid sequences and VDJ gene usage. We found that both subsets of CD8^+^ TRMs had high levels of HSV-2–specific TCRs. The levels were 2.75% and 10.20%, 6.35% and 3.64%, and 14.21% and 12.35% HSV-2–reactive clonotypes from C4, C5, and C7.1 cervix biopsy, respectively, for TRM GZMB/ITAGE^hi^ and TRM IFNG/CD69^hi^ (Table 3). These abundances are far higher than the abundance of HSV-specific CD8^+^ T cells in peripheral blood (49, 51). The results suggest that both populations of CD8^+^ TRMs in the human cervix were enriched for T cells reactive with a chronic viral pathogen that provided local intermittent antigen exposure.

## Discussion

The human ectocervix is a gateway to upper FRT and it is susceptible to several STIs, including 3 well-studied viruses: HSV, HIV, and HPV. Other viral infections, such as CMV and EBV, in the human cervix have been described (52, 53). Defining the complexities of the host immune response in this mucosal tissue is important in increasing our understanding of how the host can contain exogenous pathogens. We sought to define CD8^+^ T cell functional subsets and their TCR clonotypes in the human cervix by utilizing ectocervical biopsies and unbiased single-cell immune profiling strategies. Our data uncovered 2 major subsets of CD8^+^ TRMs in the human cervix mucosa with distinct phenotypes: one with IFNG^hi^GZMB^lo^CD69^hi^CD103^lo^LFA-1^hi^ versus the other with IFNG^lo^GZMB^hi^CD69^med^CD103^hi^LFA-1^lo^. Interestingly, TCR clonotypes of the 2 populations had little overlap. Immunofluorescence staining revealed that CD103^+^CD8^+^ TRMs were preferentially localized in the epithelium, while CD69^+^CD8^+^ TRMs were distributed evenly in the epithelium and underlying stroma. We believe that this is the first report on CD8^+^ TRM subsets with differential expression of cytolytic and noncytolytic genes and tissue-resident markers and on the association between these CD8^+^ TRM subsets with distinct expanded clonotypes and anatomic locations. By comparing both CD8^+^ TRM populations to HSV-2–reactive clonotypes from matching blood, we demonstrated that both CD8^+^ TRM populations in the human cervix may contain HSV-2–specific T cells.

CD103 and CD49a have been shown to regulate tissue residency of CD8^+^ TRMs in human and mouse skin epidermis (30, 31); LFA-1 regulates TRM tissue residency in liver and lung tissues (32–34), and CD103^+^CD69^+^ T cells have been shown to reside in human cervix tissues (11, 41, 44). We showed here that IFNG^hi^CD69^hi^ CD8^+^ TRMs expressed high levels of LFA-1 but low levels of CD103 and CD49a. In contrast, GZMB^hi^CD103^hi^ CD8^+^ TRM expressed high levels of CD103 and CD49a but low levels of LFA-1, and they were preferentially located in the epithelium. Although our studies suggest that both populations of CD8^+^ TRMs likely target HSV-2 antigens, it is unclear how the 2 distinct subsets of CD8^+^ TRMs perform immune surveillance against diverse pathogens in the human cervix. Our immunofluorescence staining analysis showed that the 2 populations of CD8^+^ TRMs had distinct anatomic locations in the cervical specimens from HSV-2–seropositive and seronegative female participants. However, our single-cell immune profiling analysis was performed in the cervical samples only from HSV-2–seropositive individuals because we did not have fresh tissues required for single-cell immune profiling in HSV-2–seronegative persons.

TRM GZMB/ITGAE^hi^ expressed high levels of CXCR3, a chemokine receptor for CXCL9 and CXCL10. Both chemokines are highly inducible in epithelial cells by IFN-γ (54, 55). We hypothesize that IFN-γ^hi^ TRMs produce IFN-γ, which induces expression of CXCL9/CXCL10 in epithelial cells, which in turn induces chemotaxis of GZMB/ITGAE^hi^ TRM to the epithelial surface of the cervix. The importance of IFN-γ in TRMs has been nicely demonstrated in mouse models for antiviral regulation and chemotaxis of other immune cells in peripheral tissues (56, 57). Assuming some of the IFN-γ^hi^ TRMs in the human cervix among HSV-2–seropositive individuals are specific for HSV-2 antigens, it would be of interest to define HSV-2 antigens targeted by these IFN-γ^hi^ TRMs, as they would be potential vaccine candidates for recurrent HSV-2 infection. In fact, a prime-pull strategy using CXCL9/CXCL10 has been nicely demonstrated to increase TRMs in the mouse vagina to control HSV infection (58).

Human TRM subsets have been demonstrated by dye efflux (22). Two recent publications have used single-cell gene expression to elucidate the subsets of small intestine intraepithelial lymphocytes (siIEL) in mouse models (23, 24). ID3^hi^Blimp1^lo^ siIEL are suggested to be stem-like TRMs. Cells in cluster 0 shown in Figure 3A have high levels of *TCF7*, *ID3,* and *ZNF683* (*Hobit*) and low levels of *PRDM1* (*Blimp1*) (data not shown), so we hypothesize that they may be stem-like TRMs. Their entire TCR repertoire (663 singletons) did not have overlap with those in cluster 7 (TRM IFNG/CD69^hi^) and only 2 of the singletons were found in cluster 1 (TRM GZMB/ITGAE^hi^) (singletons as well) (data not shown). It is unclear what the potential functions of stem-like TRMs are in the human cervix mucosa.

Single-cell gene expression analysis of purified CD8^+^ T cells clearly suggests that CD8^+^ T cells in the human cervix have a highly heterogeneous population in terms of their gene expression and clonotypes. A recent report showed that TRMs in the mouse intestine could recirculate with predilection to reestablish tissue residency in original tissues upon local reinfection (59). This study supports our observation that CD8^+^ TRMs in the human cervix may be highly dynamic in terms of their development, differentiation, and antigenic specificities.

The expanded TCR clonotypes in the human cervix are mostly from CD8^+^ T cells. Memory T cells proliferate not only in secondary lymphoid organs but also on-site in peripheral tissues (60–62). How much secondary lymphoid organs and local proliferation contribute to clonal expansion of CD8^+^ T cells in the human cervix is currently unknown. Our previous studies have shown the distinct anatomic distribution of CD8^+^ and CD4^+^ T cells in human genital skin during recurrent HSV-2 infection: CD8^+^ T cells are closer to the DEJ where HSV-2 emerges from nerve endings to infect basal keratinocytes than CD4^+^ T cells, which are found deeper in the dermal area (9, 63). This observation leads us to hypothesize that on-site proliferation likely contributes to the dominant clonotypes of CD8^+^ T cells in the human cervix.

Could we leverage CD8^+^ TRM subsets to make therapeutic vaccines against genital herpes infection? TRMs have been shown to contribute to vaccine effects against malaria in humans and tuberculosis in Rhesus macaques (64, 65). Cervical biopsies may be a useful tool to define whether such goals can be achieved. This report provides strong evidence to suggest that CD8^+^ TRM subsets are critical local immune components to perform on-site immune surveillance against infection, such as recurrent HSV-2 infection. It is therefore rational to leverage different CD8^+^ TRM subsets as vaccine targets to combat genital herpes infection and other chronic infections in the FRT.

## Methods

### Human cervix biopsy acquisition.

For 10x Genomics single-cell immune profiling, female adults (P1 to P8 in Table 1) were recruited at the University of Washington Virology Research Clinic in Seattle, Washington. The biopsy protocol was approved by University of Washington IRB committee (STUDY00004709), and all participants provided written consent. All participants were HSV-2 seropositive and HIV seronegative. Biopsy procedures were conducted as described previously (11). Biopsy procedures were performed between menstrual cycles to prevent menstrual blood in cervical samples. Biopsies (ectocervix tissue) were obtained using a baby Tischler biopsy forceps (Wallach Surgical) with a 4.2 × 2.3 mm bite size. We used lidocaine if more than one biopsy was obtained or if participants requested it (0.5 mL of 1% lidocaine injected into the cervical site prior to obtaining biopsy). The biopsy was placed in 5 mL of RPMI-CVX (RPMI 1640 medium supplemented with 5 mM L-glutamine, 50 U/mL penicillin, 50 μg/mL streptomycin, and 10% human serum, 10 μL/mL amphotericin B, and 0.5 μL/mL ciprofloxacin). All biopsies were transported to the laboratory on wet ice and processed within 4 hours of collection.

Five cervix biopsies from 4 HSV-2–seropositive participants were tested by highly sensitive PCR methods to determine whether they had HSV-2 DNA (66, 67). Three cervix biopsies were negative and 2 had very low copies of HSV-2 DNA (Supplemental Table 1).

Cervix biopsies from P9 to P15 in Table 2 were similarly obtained in the Seattle Vaccine Trials Unit at Fred Hutchison Cancer Research Center. All participants were HSV-2 and HIV seronegative, and tests for chlamydia trachomatis, Neisseria gonorrhoeae, and trichomonas vaginalis were also negative. Informed consent was obtained from all participants and was approved by Fred Hutchinson Cancer Research Center IRB (IR5640). Two ectocervical biopsies were obtained using a baby Tischler biopsy forceps (Wallach Surgical) without lidocaine. All biopsies were placed in 5 mL of RPMI-CVX, transported to the laboratory on wet ice, and processed within 4 hours of collection.

### Generation of single-cell suspension from human cervix biopsies.

Cervical biopsies were sliced into 3 to 5 strips with a sterile scalpel (size 10). The cervical strips were transferred into a sterile Falcon tube containing 3 mL of freshly prepared collagenase solution (1 mg/mL) prewarmed to 37°C (collagenase from Clostridium histolyticum, Sigma-Aldrich, C6885-500mg) and 3 μL of DNase (1U/μL, Sigma-Aldrich, DN25-10mg). Afterward, the 8 mL Falcon tube was placed in a plastic bag with a zip lock, and then placed in a 200 RPM shaker at 37°C for 30 minutes. After digestion, the biopsies were subjected to mechanical agitation by pipetting up and down 10–15 times using a 16-gauge blunt-end needle attached to a 3 mL syringe. After agitation was complete, the solution was dispensed through a 70 μm cell strainer placed on a sterile 50 mL conical tube. R15 RPMI media was used to further wash the 8 mL Falcon tube to collect any residual cells and the wash was dispensed through the 70 μm cell strainer. The R15 RPMI media wash was repeated 3–4 times until the final volume in the 50 mL conical tube reached 20 mL. The 50 mL conical tube was then centrifuged for 10 minutes at 250*g*. After centrifuging, media were removed with a serological pipette and cells were gently resuspended with 500 μL of ACK lysing buffer (Gibco, A10492-01) and incubated at room temperature for 3 minutes. After RBC lysis, 2 rounds of washing with 20 mL of 5% FBS RPMI media to neutralize ACK lysing buffer and centrifuging for 10 minutes at 250*g* were performed. After the second wash, the cell pellet was resuspended in 3 mL of R15 RPMI media, dispensed in 1 well of a 6-well culture plate (Falcon, 353046), and incubated for 1 hour in a 37°C incubator with 5% CO_2_ to allow for fibroblasts to settle and adhere. After 1 hour, cells that remained suspended in R15 RPMI media were collected and subsequently centrifuged for 10 minutes at 250*g* and washed in 1× PBS. After 2 washes in 1× PBS, cells were counted via a hemocytometer and diluted to a concentration of 1000 cells/μL for 10x Genomics processing. For CD8 negative selection, cells were lysed in ACK lysing buffer, neutralized and washed 2 times with 5% FBS RPMI media, and with or without 1-hour plating as described above, cells were further purified following the protocol from EasySep Human CD8 T Cell Isolation Kit (STEMCELL, 17953). After CD8^+^ T cells were isolated, they were washed 2 times with 1× PBS, counted via a hemocytometer, and diluted to 1000 cells/μL for 10x Genomics.

### 10x Genomics 5′ gene expression and TCR-vdj library construction.

Single cells from cervix biopsies were loaded onto the 10x Genomics Chromium Single Cell Chip. A target of 17,000 cells were loaded to account for approximately 60% cell capture rates. Cells were loaded according to 10x Genomics’ instructions for chip A to generate gel bead-in-emulsion (GEMS) using barcoded gel beads. RNA containing GEMS underwent barcoded cDNA synthesis. Subsequent library construction followed 10x Genomics 5′ single-cell immune profiling instructions. Libraries were constructed using the Chromium Next GEM Single Cell 5′ Library and Gel Beads Kit v1.1 with additional Chromium Single Cell V(D)J Enrichment Kit, Human T Cells Kit for TCR Libraries. Samples were quantified using the KAPA qPCR quantification kit and pooled equimolar for Illumina sequencing. All libraries were pooled together with a 4:1 ratio between gene expression and TCR libraries.

### Single-cell RNA-Seq data processing and analysis.

The sequencing of 10x Genomics libraries for 5′ GEX and TCR-vdj for the 10 cervix biopsies from 8 individuals and Cell Ranger analysis were performed at the shared resource at Fred Hutchison Cancer Research Center. Cell Ranger 3.0.2 with human genome GRCh38 for gene expression and GRCh38-alts-ensembl for TCR-vdj were used in the initial steps, including quality control, alignment to human genomes, and counting of aligned sequence reads for individual genes. We manually examined summary HTML files for the 10 gene expression libraries and the 10 TCR-vdj libraries generated from 10 cervix samples. All the libraries had valid barcodes (>85%), fraction reads in cells (>80%), and reads mapped to genome (>50%). The summary of the number of cells sequenced and number of cells with TCR-vdj data for the 10 cervix samples is described in Table 1. The initial analysis was performed in 10x Genomics’ cell Loupe browser and vdj Loupe browser. The MTX files from the output of Cell Ranger were imported into Monocle3 (68), Seurat (version 3.1.2) (69), or Scanpy (version 1.4.4) (70) for further analysis. In all 3 methods, we filtered out cells with less than 200 genes detected and genes only detected in less than 3 cells. We further filtered out cells with percentages of mitochondria genes of more than 15%. pyVDJ (https://github.com/veghp/pyVDJ) and Scirpy (71) were used to link TCR-vdj to gene expression in Scanpy. To perform unsupervised clustering analysis of single-cell gene expression data, we used UMAP (uniform manifold approximation and projection) or tSNE (t-distributed stochastic neighbor embedding) as dimension reduction methods and Leiden (an algorithm for methods of community detection in large networks) as the default clustering method. Trajectory analysis described in Figure 3 was performed in Monocle3. For the analysis described in Figure 2, batch correction among the 8 cervical samples from the 6 participants using BBKNN methods in Scanpy were performed before clustering analysis (72).

### Immunofluorescence staining and image analysis.

The immunofluorescence staining was performed as previously described (9, 73). Cervical biopsies were cut in half and embedded in Tissue-Tek OCT (Sakura Finetek, Thermo Fisher Scientific) and immediately frozen in 2-methylbutane on dry ice and stored at –80°C. Frozen tissue blocks were cut into 8 μm sections and stored at –80°C. Slides were thawed and fixed in acetone for 15 minutes at –20°C and then left to dry for 30 minutes at room temperature. Slides were then quenched in 3% H_2_O_2_ for 60 minutes at room temperature and then in blocking buffer (PBS containing 5% normal human and goat sera) for 1 hour followed by an incubation of primary antibodies (anti-CD69 or anti-CD103). Anti-CD69 (eBioscience, 14-0699-82) was done followed by the TSB goat anti-mouse protocol (Thermo Fisher Scientific, B40912), and CD8-AF647 (BD Biosciences, 557708) was stained ON at 4°C in block solution. Anti-CD103 (Abcam, 224202) was done followed by the TSB goat anti-rabbit 555 protocol (Thermo Fisher Scientific, B40923) and CD8-AF647 was stained ON at 4°C in block solution. Cell nuclei were counter-stained with DAPI (Thermo Fisher Scientific, D3571). Slides were mounted with Prolong Gold (Invitrogen, P36930) and cured at least 24 hours prior to imaging.

Images were analyzed in Fiji, an open-source image processing package based on ImageJ (NIH). Areas of the epithelium and stroma were analyzed separately. The image was then split into DAPI + CD69 and DAPI + CD8. Each cell type was counted in the epithelium and stroma separately using Cell Counter. An all-color combined image was counted for double-positive cells in each area. The same analysis was done for CD103 + CD8 images.

### Combination of FISH and IHC staining.

Fresh, frozen cervix biopsies were cryosectioned into 10 mm slides and fixed with 4% paraformaldehyde and dehydrated in ethanol. Dehydrated slides were pretreated by hydrogen peroxide and 0.3% Triton X-100 before FISH probe hybridization using RNAscope multiplex fluorescent reagent kit v2 (Advanced Cell Diagnostics, 323100). FISH Probes included IFNG and GZMB (Advanced Cell Diagnostics, 3105 and 31051_C2 for IFNG and 445971 for GZMB). Probe hybridized slides were washed by washing buffer (Advanced Cell Diagnostics, 323100) before IHC using AF488 tyramide SuperBoost kit (Invitrogen, B40912). Slides were incubated with block buffer for 60 minutes at room temperature and then with primary antibodies for 60 minutes at room temperature. Primary antibodies included CD69 (eBioscience, 14-0669-82) and CD103 (Abcam, ab224202). After washing with PBS with 0.1% Tween 20, the slides were incubated with poly-HRP–conjugated secondary antibodies for 60 minutes at room temperature. Slides were washed with 1× PBS with 0.1% Tween 20 and incubated with tyramide for 5 minutes at room temperature. Slides were counterstained with DAPI (Thermo Fisher Scientific, D3571) and mounted in Prolong Gold Antifade Mountant (Thermo Fisher Scientific, P36930).

### Definition of blood CD8^+^ TCR clonotypes reactive with HSV-2.

HSV-2–specific CD8^+^ T cells were identified and isolated using a method similar to that previously reported for HSV-1 (49). In brief, PBMCs were cryopreserved from venous anticoagulated blood using standard methods; 2 × 10^5^ monocyte-derived DCs — generated from adherent PBMCs in the presence of IL-4 and GM-CSF as described (74) — were incubated for 4 hours at 37°C/5% CO_2_ with 2 × 10^5^ UV-irradiated (Stratalinker XL1000, 180,000 μJ) HeLa cells that had either been infected overnight with HSV-2 strain 186 (MOI 2.5) or left uninfected (mock). Autologous CD8^+^ T cells were negatively selected (STEMCELL EasySep Human CD8 T Cell Isolation Kit) and incubated at 1 × 10^6^ to 2 × 10^6^ T cells per well with the monocyte-derived DCs/HeLa mix. PHA-P (1.6 μg/mL, Remel) was used as a positive control. T cells were harvested after 18–20 hours of incubation and stained with anti–CD3-ECD (UCHT1, Beckman Coulter), anti–CD8-FITC (3B5, Thermo Fisher Scientific), anti–CD137-APC (4B4-1, BD Biosciences), and 7-AAD (BD Biosciences). Live (7-AAD negative), single CD3^+^CD8^+^ lymphocytes that expressed CD137 were sorted using a BD Biosciences FACS Aria II (University of Washington Cell Analysis Facility), pelleted, and stored at –80°C. DNA was extracted from cell pellets using the DNeasy blood and tissue kit (QIAGEN) following the manufacturer’s instructions, which was submitted to Adaptive Biotechnologies for TCRB sequencing.

### Data deposit.

The raw data for single-cell gene expression and TCR clonotypes were deposited in NCBI’s Gene Expression Omnibus (GEO GSE173231). The TCRB data for blood CD137^+^CD8^+^ cells described in Table 3 are available here: https://clients.adaptivebiotech.com/pub/peng-2021-jcii

### Statistics.

Two-sample 2-tailed *t* tests were performed to derive the *P* values described in Figures 2, 4, and 6. *P* values of less than 0.05 were considered significant.

### Study approval.

Written informed consent was received from participants prior to inclusion in the study. As described in Tables 1 and 2, the cervix specimens (C1 to C8) were obtained at University of Washington Virology Research Clinic with IRB approval (STUDY00004709). The cervix specimens (C9 to C15) were obtained at the Seattle Vaccine Trials Unit at Fred Hutchison Cancer Research Center with IRB approval (IR5640).

## Author contributions

LC, JZ, and TP conceived the study. TP did the analysis and wrote the paper. KP did the tissue digestion, and EB and ANL from JB’s laboratory did the 10x Genomics library construction. AK and LJ did immunofluorescence staining. CJ, AW, and DV performed the cervix biopsy procedures at University of Washington. NAW and JC from MJM’s group prepared the cervix biopsy at Fred Hutchison Cancer Research Center. DK and KL isolated HSV-2–reactive CD8^+^ T cells from PBMCs. LC, JZ, DK, and KL contributed to writing the paper.

## Supplementary Material

Supplemental data

## Figures and Tables

**Table 2:**
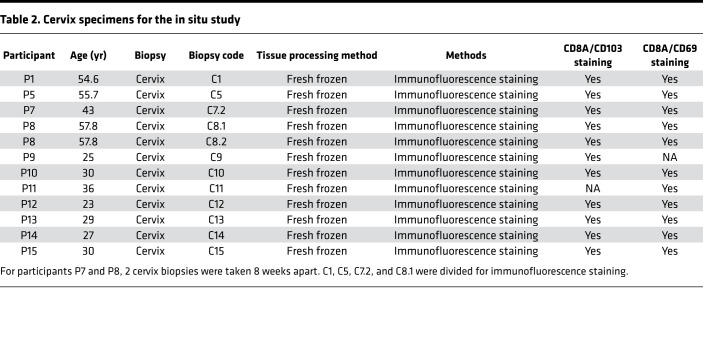
Cervix specimens for the in situ study

**Table 1:**
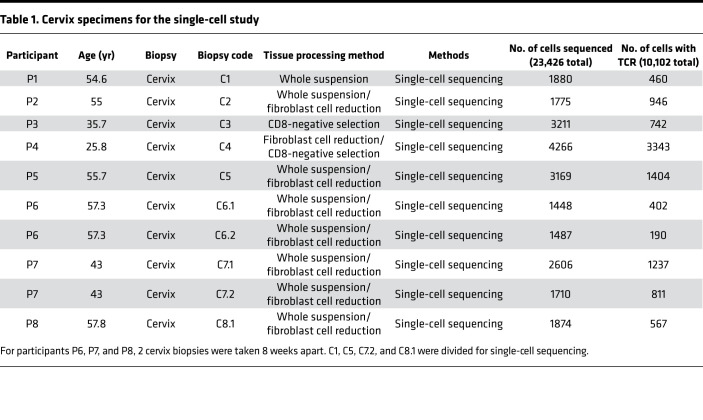
Cervix specimens for the single-cell study

**Table 3:**
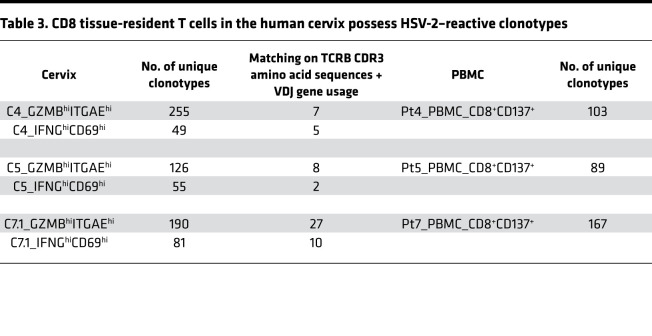
CD8 tissue-resident T cells in the human cervix possess HSV-2–reactive clonotypes

## Version 1 06/22/2021

In-Press Preview

## Version 2 08/09/2021

Electronic publication
