# Supplementary material for: Distinct populations of antigen-specific tissue-resident CD8+ T cells in human cervix mucosa
Source: JCI Insight. 2021 Aug 9;6(15):e149950. doi: 10.1172/jci.insight.149950 (PMC8410090; doi:10.1172/jci.insight.149950)
Supplement: Supplemental data [file jciinsight-6-149950-s215.pdf]

Supplementary Table 1

Cervix biopsy code	Target Name	Copies/reaction	Target Name	Copies/reaction
C8.1	HSV	4.43	beta globin	4,396.93
C8.2	HSV	Undetectable	beta globin	2,784.81
C7.2	HSV	2.35	beta globin	3,932.73
C5	HSV	Undetectable	beta globin	4,613.60
C1	HSV	Undetectable	beta globin	6,074.39

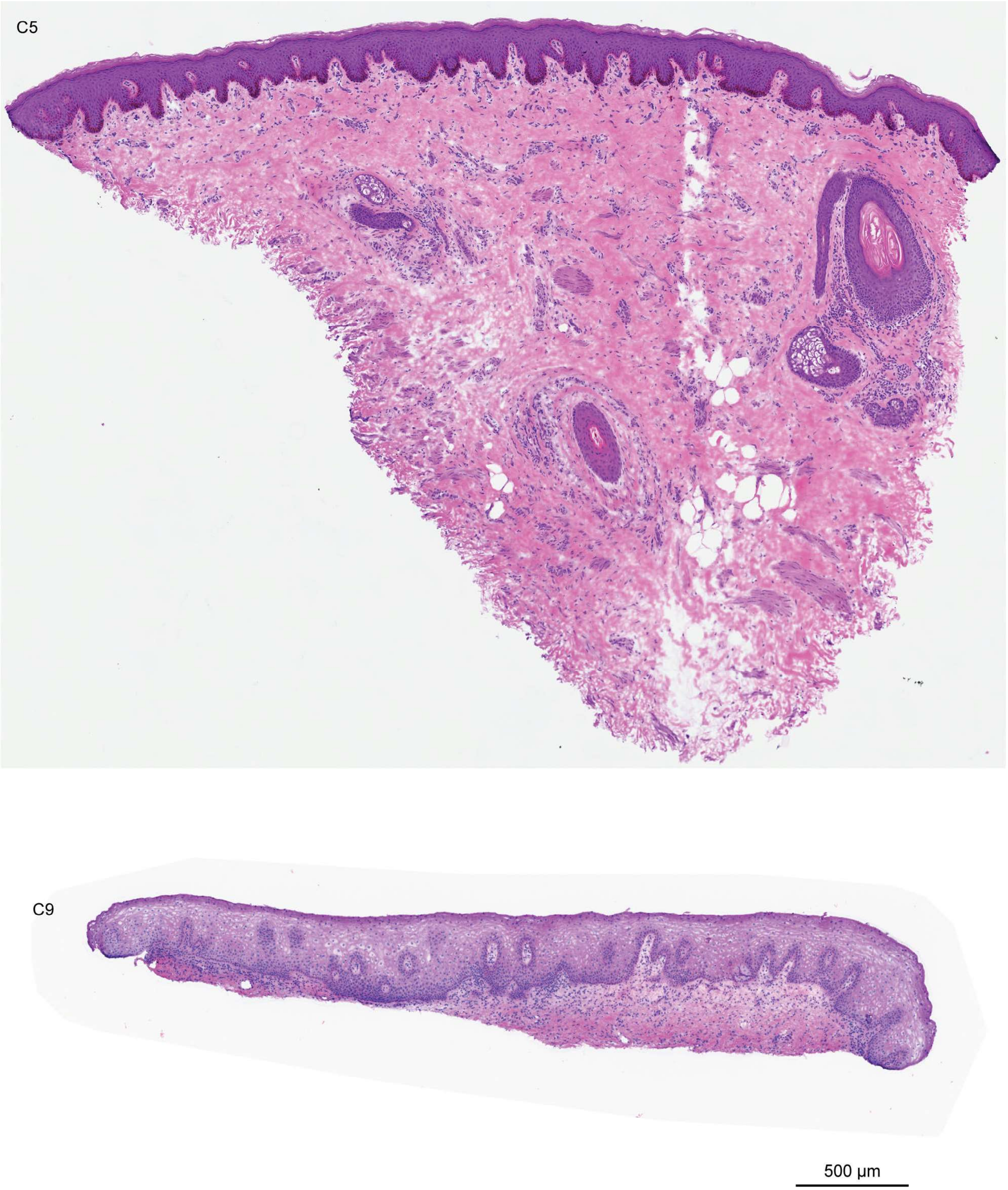

Supplementary Figure 1. H&E staining of the human cervix (C5 and C9).

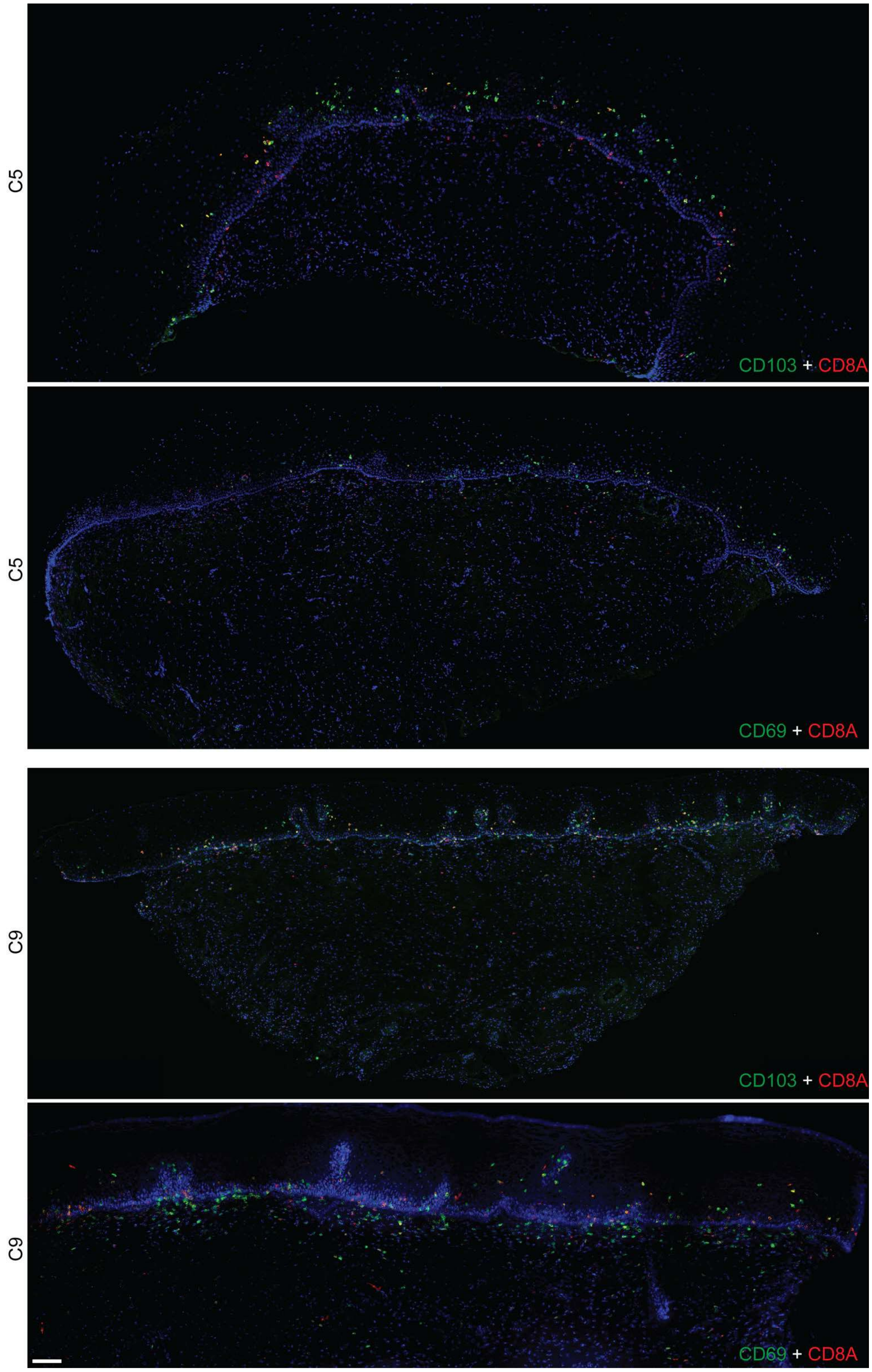

Supplementary Figure 2. Double immunofluorescent staining of cervix biopsies (C5 and C9) with CD8A and CD103 antibodies, or CD8A and CD69 antibodies. Images for the whole slides were shown. Scale bar = 100 um.
